# Effects of Prolonged Head-Down Bed Rest on Cardiac and Vascular Baroreceptor Modulation and Orthostatic Tolerance in Healthy Individuals

**DOI:** 10.3389/fphys.2019.01061

**Published:** 2019-08-23

**Authors:** Franca Barbic, Karsten Heusser, Maura Minonzio, Dana Shiffer, Beatrice Cairo, Jens Tank, Jens Jordan, André Diedrich, Peter Gauger, Roberto Antonio Zamuner, Alberto Porta, Raffaello Furlan

**Affiliations:** ^1^Humanitas Clinical and Research Center, Department of Internal Medicine, Istituto di Ricovero e Cura a Carattere Scientifico (IRCCS), Humanitas University, Rozzano, Italy; ^2^German Aerospace Center (DLR), Institute of Aerospace Medicine, Cologne, Germany; ^3^Department of Biomedical Sciences for Health, University of Milan, Milan, Italy; ^4^Autonomic Dysfunction Center, Clinical Research Center (CRC), Department of Medicine, Vanderbilt University, Nashville, TN, United States; ^5^Departamento de Kinesiología, Universidad Católica del Maule, Talca, Chile; ^6^Department of Cardiothoracic, Vascular Anesthesia and Intensive Care, Istituto di Ricovero e Cura a Carattere Scientifico (IRCCS) Policlinico di San Donato, San Donato Milanese, Italy

**Keywords:** orthostatic intolerance, bed rest, baroreflex sensitivity, muscle sympathetic nerve activity, spectrum analysis

## Abstract

Orthostatic intolerance commonly occurs after prolonged bed rest, thus increasing the risk of syncope and falls. Baroreflex-mediated adjustments of heart rate and sympathetic vasomotor activity (muscle sympathetic nerve activity – MSNA) are crucial for orthostatic tolerance. We hypothesized that prolonged bed rest deconditioning alters overall baroreceptor functioning, thereby reducing orthostatic tolerance in healthy volunteers. As part of the European Space Agency Medium-term Bed Rest protocol, 10 volunteers were studied before and after 21 days of −6° head down bed rest (HDBR). In both conditions, subjects underwent ECG, beat-by-beat blood pressure, respiratory activity, and MSNA recordings while supine (REST) and during a 15-min 80° head-up tilt (TILT) followed by a 3-min −10 mmHg stepwise increase of lower body negative pressure to pre-syncope. Cardiac baroreflex sensitivity (cBRS) was obtained in the time (sequence method) and frequency domain (spectrum and cross-spectrum analyses of RR interval and systolic arterial pressure – SAP, variability). Baroreceptor modulation of sympathetic discharge activity to the vessels (sBRS) was estimated by the slope of the regression line between the percentage of MSNA burst occurrence and diastolic arterial pressure. Orthostatic tolerance significantly decreased after HDBR (12 ± 0.6 min) compared to before (21 ± 0.6 min). While supine, heart rate, SAP, and cBRS were unchanged before and after HDBR, sBRS gain was slightly depressed after than before HDBR (sBRS: −6.0 ± 1.1 versus −2.9 ± 1.5 burst% × mmHg^−1^, respectively). During TILT, HR was higher after than before HDBR (116 ± 4 b/min versus 100 ± 4 b/min, respectively), SAP was unmodified in both conditions, and cBRS indexes were lower after HDBR (*α* index: 3.4 ± 0.7 ms/mmHg; BRS_SEQ_ 4.0 ± 1.0) than before (*α* index: 6.4 ± 1.0 ms/mmHg; BRS_SEQ_ 6.8 ± 1.2). sBRS gain was significantly more depressed after HDBR than before (sBRS: −2.3 ± 0.7 versus −4.4 ± 0.4 burst% × mmHg^−1^, respectively). Our findings suggest that baroreflex-mediated adjustments in heart rate and MSNA are impaired after prolonged bed rest. The mechanism likely contributes to the decrease in orthostatic tolerance.

## Introduction

In 1944, Dock pointed out that “The physician must always consider complete bed rest as a highly un-physiologic and definitely hazardous form of therapy, to be ordered only for specific indications and discontinued as early as possible” (Dock, 1944). The statement, which challenged medical beliefs of that period, is now supported by numerous physiological investigations and clinical observations. For example, bed rest is associated with reductions in both effective circulating blood volume and cardiac output. Moreover, muscular atrophy particularly of lower limbs, thromboembolism, and infections may occur (Allen et al., 1999; McIntyre, 2013). In addition, prolonged bed rest predisposes to the common hospitalization-associated disability syndrome (Allen et al., 1999; Covinsky et al., 2011; Ettinger, 2011). Orthostatic intolerance and syncope have been observed after prolonged bed rest in various clinical settings (Feldstein and Weder, 2012; Guerin et al., 2016; Tzur et al., 2018). The condition negatively impacts patients’ quality of life and increases the risk of falls (Shibao et al., 2007; Juraschek et al., 2017). Sometimes dramatic impairments in orthostatic tolerance have also been observed in astronauts returning to Earth (Ertl et al., 2002; Levine et al., 2002; Diedrich et al., 2007, 2015), which led to the discovery of novel mechanisms affecting orthostatic tolerance (Levine et al., 1997; Diedrich et al., 2007). For example, weightlessness elicited changes in muscle sympathetic nerve activity (MSNA) (Ertl et al., 2002) and in baroreflex heart rate regulation (Cox et al., 2002; Eckberg et al., 2010).

In healthy humans, venous pooling below the heart upon standing tends to reduce cardiac output and blood pressure (Mosqueda-Garcia et al., 1997; Furlan et al., 2001; Diedrich and Biaggioni, 2004). These changes unload cardiopulmonary and arterial baroreceptors eliciting compensatory changes in heart rate (HR) and in muscle sympathetic nerve activity (MSNA) (Mosqueda-Garcia et al., 1997; Furlan et al., 2000; Barbic et al., 2015). In healthy subjects, HR, plasma norepinephrine, and MSNA markedly increase with standing. Thus, systolic blood pressure is maintained while diastolic blood pressure slightly increases (Furlan et al., 2000). Conversely, an impaired baroreflex function as observed in patients with baroreflex failure (Robertson et al., 1993; Furlan et al., 2001; Heusser et al., 2005) promotes orthostatic intolerance. In addition, a proper baroreceptor function plays a crucial role in synchronizing the neural sympathetic discharge activity and the cardiovascular spontaneous fluctuations at 0.1 Hz (LF) in the upright position (Furlan et al., 2000). The synchronization appears to be important for orthostatic tolerance (Furlan et al., 2000, 2015; Barbic et al., 2007).

We tested the hypothesis that a controlled long-lasting bed rest may induce changes in baroreceptor response while supine and during up-right position, eventually resulting in reduced orthostatic tolerance in healthy volunteers.

## Materials and Methods

### Experimental Protocol

As part of the European Space Agency Medium-Term-Bed Rest Study (ClinicalTrials.gov Identifier: NCT01655979; Buehlmeier et al., 2014), 10 healthy men (33 ± 1 years, BMI 23.4 ± 0.2 kg/m^2^) were studied before and after 21 days of −6° head down bed rest (HDBR). The study was conducted at the DLR facilities of the Institute of Aerospace Medicine (Colonie, Germany).

Before starting with the study protocol, the subjects were confined to the metabolic ward of the German Aerospace Center for 7 days for environmental, routine, and diet adaptation. During the intervention period (HDBR), all the activities of daily routine such as eating and hygienic procedures took place in bed. The subjects were allowed to change their horizontal position by maintaining at least one shoulder in contact with the mattress. Muscular activity of the legs was not allowed. A passive physical therapy was included regularly every 3–4 days to reduce the psychological tension. The adherence to the study rules was controlled by study nurses in charge and by a continuous 24-h video monitoring (Buehlmeier et al., 2014).

During the HDBR, volunteers were encouraged to keep a constant day and night routine, characterized by 16–17 h of wakefulness and 7–8 h of night sleep. Ward lights were turned off from 11 pm to 6 am. Temperature and humidity inside the metabolic ward were controlled during the study (21.5 ± 1°C, 40 ± 6.3%) (Buehlmeier et al., 2014).

An independent medical doctor monitored the subjects’ health status during daily ward rounds. No adverse events according to good clinical practice were reported, and no drugs potentially affecting cardiovascular autonomic system were prescribed during HDBR (Buehlmeier et al., 2014).

Diet composition followed the requirements given by a standardization document of ESA (“Standardization of bed rest study conditions,” Version 1.5) based on 120% of resting metabolic rate (RMR) to account for the low physical activity associated with the bed rest period (Buehlmeier et al., 2014). Methyl-xanthine derivatives (e.g., caffeine), alcohol, and flavor enhancers were prohibited.

During the HDBR, the volunteers were supplemented with 1,000 IU vitamin D3 per day in order to overcome the sunlight exclusion (Buehlmeier et al., 2014).

Before and after 21 days of −6° head down bed rest (HDBR), all subjects underwent to continuous ECG, beat-by-beat blood pressure (BP; Finapres Medical Systems, Ohmeda), respiratory rate (Electrobioimpedance Amplifier, Biopac System, Inc.), and MSNA (Nerve Traffic Analyzer; model 662C-3; University of Iowa Bioengineering, Iowa City, IA, USA) recordings. Measurements were obtained in the supine position (REST) and during 15 min of 80° head-up tilt (TILT) followed by a 3-min −10 mmHg stepwise increase of lower body negative pressure (LBNP) up to pre-syncope. Pre-syncope was defined as progressive hypotension, tachycardia/bradycardia, pallor, yawning, and symptoms including sweating, nausea, and lightheadedness (el-Bedawi and Hainsworth, 1994; Protheroe et al., 2013). Tilt termination criteria were as follows: sudden onset of pallor, blurred vision, lightheadedness, sweating, nausea, an increase or a decrease in HR greater than 40% and/or a decrease in systolic arterial pressure (SAP) greater than 40% compared to what observed during the first 5 min of asymptomatic TILT.

The time of TILT to pre-syncope was computed to quantify the orthostatic tolerance.

MSNA was recorded from the peroneal nerve of the right leg as detailed elsewhere (Mosqueda-Garcia, 1996). Briefly, multiunit recordings of postganglionic sympathetic activity were obtained by placing a tungsten electrode in the right peroneal nerve, posterior to the fibular head. A reference electrode was inserted subcutaneously, close by the recording needle.

This study was carried out in accordance with the recommendations of the Aerztekammer Nordrhein (Dusseldorf, Germany) with written informed consent from all subjects. The protocol was approved by the ethic committee of the Aerztekammer Nordrhein (Dusseldorf, Germany).

### Data Analysis

ECG, BP, respiratory activity, and MSNA were digitized at 500 Hz by an analog-to-digital converter (AT-MIO 16E2; National Instruments) and recorded with BNC-2110 data acquisition system and LabVIEW 7.0 software (National Instruments, Austin, TX, USA) for off-line analysis.

MSNA raw signal was filtered (700–2,000 Hz), amplified (1,000 × 99.9), rectified, and integrated with a time constant of 0.1 s *via* a nerve traffic analysis system (662C-3, University of Iowa). The sympathetic bursts were detected by an adaptive thresholding methodology accounting for the baseline wandering and different MSNA burst amplitudes as previously described (Diedrich et al., 2009). Specifically, the burst detection threshold was updated on a beat-to-beat basis to follow baseline wandering and changes of MSNA burst amplitude (Diedrich et al., 2009). The threshold was then assessed by calculating the minimum value of the sympathetic burst and the difference between the maximum and minimum values in each cardiac cycle. The running threshold was provided by the minimum value plus 30% of the difference between the maximum and minimum values of the burst.

The MSNA burst was searched in a temporal window ranging from 0.9 to 1.7 s starting from the R-wave peak of the first R-wave peak delimiting the current cardiac cycle to account for the latency from aortic and carotid baroreceptor stimulation to the potential vascular sympathetic response (Macefield et al., 1994; Wallin et al., 1994; Diedrich et al., 2009). SAP was computed as the maximum BP in a given heart period approximated as the temporal distance between two successive R-wave peaks detected in the ECG. Diastolic arterial pressure (DAP) was computed as the minimum arterial pressure following SAP. The temporal occurrences of the MSNA burst and DAP were also stored.

Autoregressive spectrum and cross-spectrum analysis of RR interval, SAP, and respiratory activity variability have been described in detail elsewhere (Pagani et al., 1986; Furlan et al., 2000; Barbic et al., 2007). For RR interval spontaneous variability, there are two major spectral components, the amplitude of which is affected by changes in cardiac neural autonomic control (Pagani et al., 1986; Furlan et al., 2000; Barbic et al., 2007). One is the high frequency, HF component (HF_RR_, 0.25 Hz), synchronous with the respiration, an accepted index of vagal modulation to the sinoatrial node (Task Force of the European Society of Cardiology and the North American Society of Pacing and Electrophysiology, 1996; Furlan et al., 2000; Barbic et al., 2007). The other is the low frequency, LF (LF_RR_, 0.1 Hz), that when expressed in normalized units has been proposed to primarily reflect the sympathetic efferent modulation to the sinoatrial node and its changes (Pagani et al., 1986; Task Force of the European Society of Cardiology and the North American Society of Pacing and Electrophysiology, 1996; Furlan et al., 2000; Barbic et al., 2007). Spectral components of RR variability in the high frequency (HF) and in the low frequency (LF) range are provided in absolute (ms^2^) and in normalized units (n.u.). Absolute values of each component were computed as the integral of the oscillatory components LF_RR_ and HF_RR_. Normalization was achieved by dividing the absolute power of each component by total variance minus the power of the very-low frequency component (0.03 Hz) and subsequently multiplying by 100 (Task Force of the European Society of Cardiology and the North American Society of Pacing and Electrophysiology, 1996). The LF/HF is a dimensionless index of the instantaneous reciprocal changes of cardiac sympathetic and vagal modulation (Pagani et al., 1986; Task Force of the European Society of Cardiology and the North American Society of Pacing and Electrophysiology, 1996). The LF oscillatory component of SAP variability (LF_SAP_, 0.1 Hz), expressed in absolute values, is a marker of the sympathetic vascular modulation (Furlan et al., 2000; Barbic et al., 2007).

The time series length of REST, and TILT comprised of 300 consecutive beats, recorded 3 min before tilt interruption because of pre-syncope. The stationarity of the identified sequence was tested according to Magagnin and colleagues (Magagnin et al., 2011) over the original series after linear de-trending. If the test for the steadiness of mean and variance was not satisfied, a new selection was identified to have all the prerequisites for guaranteed restricted weak stationarity (Magagnin et al., 2011). Indeed, the stationarity of the mean is necessary even after linear de-trending because the cardiovascular variability trends are complex and not completely addressed by a simple linear approach.

All the analyses were performed on signals recorded in supine position (REST) and after 3 min of head-up tilt when all the volunteers were asymptomatic (TILT) before and after HDBR.

### Baroreflex Control of Heart Rate

The cardiac baroreflex sensitivity (cBRS) was obtained in the frequency domain by the alpha index (*α*); Pagani et al., 1986; Furlan et al., 2000) and in the time domain according to the baroreflex sequence analysis approach (Bertinieri et al., 1988; Parati et al., 1988) as previously implemented by Porta et al. (2013).

The frequency domain approach is based on cross-spectral analysis of RR and SAP variability. After having obtained a squared coherence function (*K*^2^) >0.5, the *α* index was computed as the square root of the ratio between the powers of the LF (0.1 Hz) spectral components of RR interval and SAP variability (Pagani et al., 1988).

The sequence analysis approach is based on the search for sequences characterized by the contemporaneous increase (positive sequence) or decrease (negative sequence) of RR and SAP values. Both positive and negative sequences are referred to as baroreflex sequences as previously described (Bertinieri et al., 1988). They were identified according to the following prerequisites: (1) the length of the sequences was four beats (three increases or decreases); (2) the lag between RR and SAP values was set to 0; (3) the total SAP variation was larger than 1 mmHg; (4) the total RR variation was larger than 5 ms; and (5) the correlation coefficient in the plane [SAP(i), RR(i)], where (i) is the cardiac beat number, was larger than 0.85. When a baroreflex sequence matched those prerequisites, the slope of the regression line in the plane [SAP(i), RR(i)] was calculated and averaged over all baroreflex sequences. This average was indicated as BRS and expressed as ms/mmHg. The percentage of baroreflex sequences found in the analyzed signals was also quantified.

### Baroreflex Control of Sympathetic Activity to the Vessels

**Figure 1:**
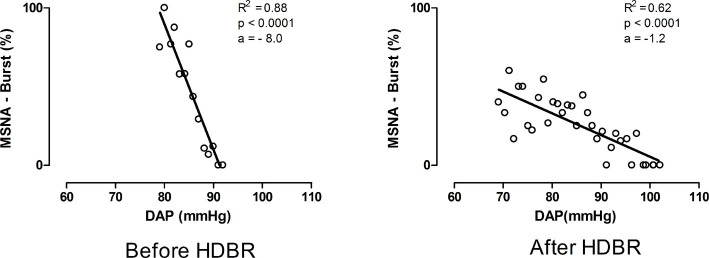
Representative example of the values of sBRS during 80° head-up tilt before and after bed rest. Notice that after bed rest the gain of sBRS indicated by the slope (*a*) of the regression line between MSNA burst % occurrence and DAP values was lower as reflected by a flatter regression line than pre HDBR.

The assessment of sBRS considers how the DAP value relates to the occurrence of a MSNA burst accounting for the baroreflex latency (Hart et al., 2010). As previously described (Hart et al., 2010; Barbic et al., 2015; Marchi et al., 2015), DAP values were grouped into bins of 1 mmHg; the percentage of times that a MSNA burst was detected as associated with the considered values of DAP was counted. A weighted linear regression between nerve activity and DAP was performed. In the plane reporting MSNA burst incidence values (%) on the *y* axis and DAP values on the *x* axis, a linear regression analysis was performed. The slope of the regression line (*a*) furnished the index of sBRS gain provided that the correlation coefficient (*r*_sBRS_) was significant (*p* < 0.05). The slope of the regression line is a negative value. Therefore, the steeper the sBRS gain, the more negative is the slope (Figure 1). Conversely, when the slope tends to 0, the regression line is flatter and the sBRS gain is less negative. More negative values correspond to a more efficient sympathetic baroreflex, while less negative value to a more depressed sympathetic baroreflex. Indeed, flattening of the DAP-MSNA relationship implies a decrease in the sympathetic modulation to the vessels in response to a unit change of DAP (Barbic et al., 2015).

### Statistical Analysis

Continuous variables are expressed as mean ± standard error. The normality of data was tested *via* Kolmogorov-Smirnov test. Paired *t* test was used to assess differences in orthostatic tolerance time before and after HDBR. Repeated measure two-way analysis of variance followed by Holm-Sidak *post hoc* test was used to assess differences in hemodynamics, respiration cardiovascular autonomic parameters, baroreflex control indexes, and MSNA between REST and TILT before and after HDBR. The level of significance was set at 5%. SigmaPlot 11 (Systat Software Inc., Chicago, IL, USA) was used for statistical analysis.

## Results

**Figure 2:**
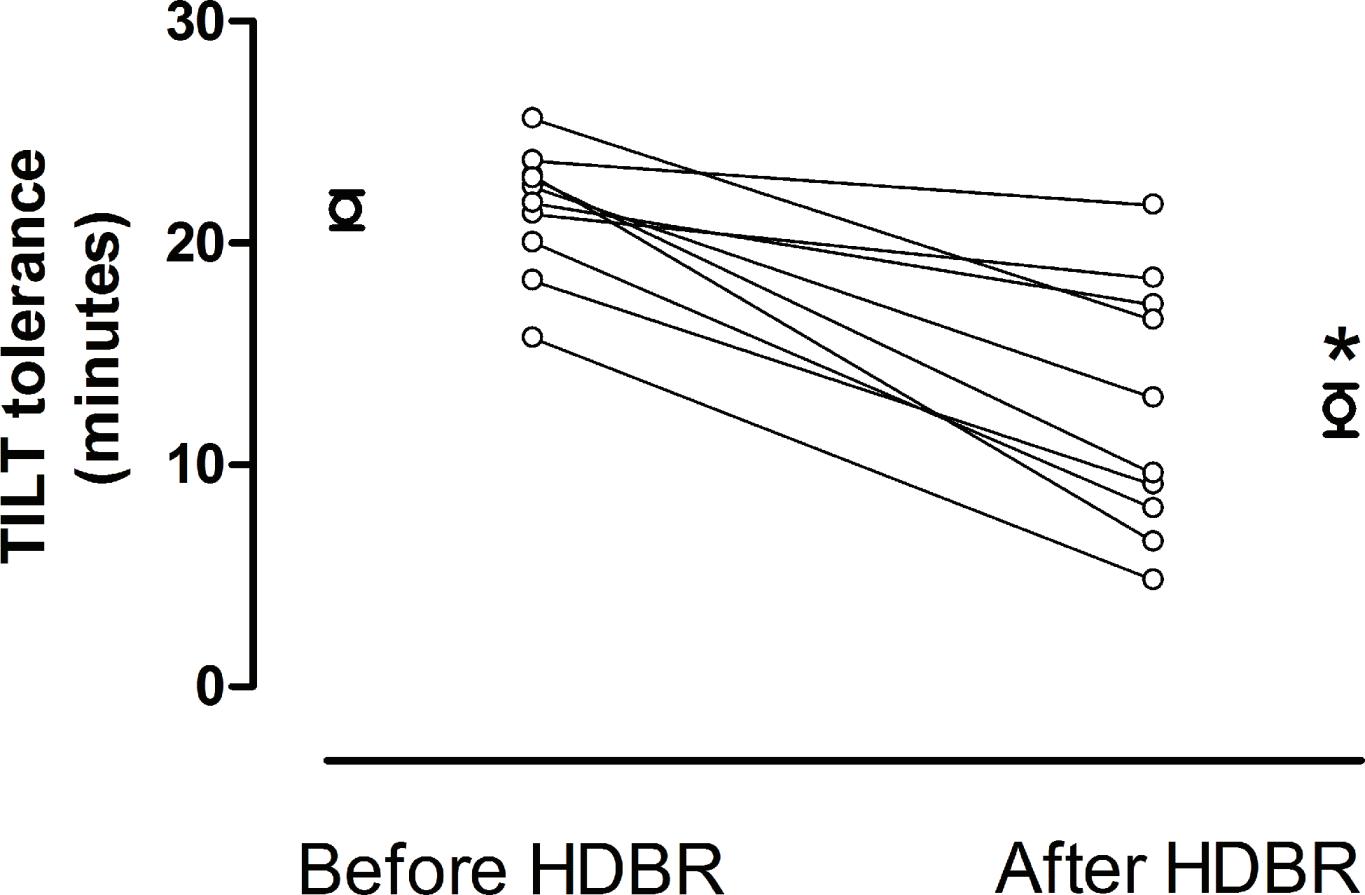
Individual and mean values ± standard error of the 80° head-up tilt tolerance before and after HDBR. After HDBR orthostatic tolerance was significantly reduced compared to before HDBR.

The time to pre-syncope during orthostatic testing before and after HDBR is shown in Figure 2. The mean time to pre-syncope was 21.5 ± 0.8 min before and 12.5 ± 1.1 min after HDBR (*p* < 0.05). Before HDBR, all subjects required additional LBNP application to induce pre-syncope. Three subjects experienced pre-syncope with −10 mmHg, one with −20 mmHg, five with −30 mmHg, and one with −40 mmHg of LBNP. Conversely, after HDBR only in three of 10 volunteers, a −10 mmHg of LBNP was necessary to induce pre-syncope.

The mean values of the hemodynamics and respiratory activity while supine (REST) and during TILT before and after HDBR are reported in Table 1.

**Table 1:** Hemodynamics and respiratory activity while supine (REST) and during 80° head-up tilt (TILT) before and after HDBR.

	Before HDBR		After HDBR	
Parameters	REST	TILT	REST	TILT
HR, b/min	71.8 ± 3.0	99.7 ± 4.1^*^	70.7 ± 2.2	116.1 ± 4.1^*^^,^^#^
SAP, mmHg	130 ± 4	129 ± 4	130 ± 3	129 ± 6
DAP, mmHg	76 ± 2	88 ± 3^*^	74 ± 2	87 ± 3^*^
RESP, breaths/min	16.5 ± 1.9	16.2 ± 0.9	14.6 ± 0.5	15.4 ± 1.3

* *REST vs. TILT *p* < 0.05*.

# *Before HDBR vs. after HDBR *p* < 0.05*.

*HR, heart rate; SAP, systolic arterial pressure; DAP, diastolic arterial pressure; RESP, respiratory frequency; MSNA, muscle sympathetic nerve activity. Values are expressed as mean ± standard error*.

Before HDBR, HR and DAP values were higher during TILT than during REST, whereas no differences were observed in the SAP and respiration. After HDBR, HR and DAP were greater during TILT than during REST, without modifications in both SAP and respiratory activity (Table 1). Notably, HR during tilt after HDBR was greater than before HDBR (Table 1).

Cardiovascular autonomic as well as cardiac and sympathetic baroreflex indexes while supine (REST) and during TILT before and after HDBR are shown in Table 2.

**Table 2:** Autonomic parameters, cardiac and sympathetic baroreceptor indexes, and MSNA assessed while supine (REST) and during 80° head-up tilt (TILT), before and after HDBR.

	Before HDBR		After HDBR	
Parameters	REST	TILT	REST	TILT
RR, ms	848 ± 35	634 ± 25^*^	856 ± 26	522 ± 19^*^^,^^#^
RR var., ms^2^	2,288 ± 279	1,632 ± 412	2,270 ± 497	465 ± 176^*^^,^^#^
SAP var., mmHg^2^	11.9 ± 2.1	40.5 ± 6.6^*^	11.8 ± 1.8	39.3 ± 10.7^*^
LF_RR_, ms^2^	805 ± 177	1,167 ± 298	649 ± 145	235 ± 82^#^
n.u.	58.7 ± 6.6	87.6 ± 3.4^*^	67.8 ± 6.1	83.5 ± 3.5^*^
HF_RR_, ms^2^	390 ± 85	154 ± 65^*^	347 ± 121	28 ± 12^*^
n.u.	36.7 ± 7.4	11.0 ± 3.2^*^	31.0 ± 6.1	14.7 ± 3.4^*^
LF/HF	2.7 ± 0.4	19.2 ± 2.1	3.1 ± 0.2	17.1 ± 3.1
LF_SAP_, mmHg^2^	5.4 ± 1.0	32.3 ± 6.6^*^	4.1 ± 1.1	23.9 ± 7.7^*^
*α*, ms/mmHg	12.4 ± 0.9	6.4 ± 1.0^*^	14.5 ± 1.9	3.4 ± 0.7^*#^
cBRS_SEQ_	18.1 ± 1.9	6.8 ± 1.2^*^	15.3 ± 2.7	4.0 ± 1.0^*^
sBRS^§^, burst% × mmHg^−1^	−6.0 ± 1.1	−4.4 ± 0.4	−2.9 ± 1.5^#^	−2.3 ± 0.7^#^
MSNA^§^, burst/min	18.0 ± 1.5	29.1 ± 1.7^*^	25.3 ± 1.8^#^	32.2 ± 2.3
MSNA^§^, burst/100 beats	26.9 ± 2.5	30.4 ± 2.4	35.0 ± 2.7	27.8 ± 2.7

§ *MSNA, *n* = 8 subjects*.

* *REST vs. TILT *p* < 0.05*.

# *Before HDBR vs. after HDBR, *p* < 0.05*.

*RR, RR interval; var., variance; SAP, systolic arterial pressure; LF, low frequency; n.u., normalized units; HF, high frequency; *α*, cardiac baroreflex index assessed in the frequency domain; cBRS_SEQ_, cardiac baroreflex index assessed in the time domain; sBRS, sympathetic baroreflex gain; MSNA, muscle sympathetic nerve activity*.

At REST, cardiac baroreceptor control indices *α* and cBRS were unchanged after HDBR. The spectral indices of cardiac sympathetic and vagal modulation were only slightly modified, namely an increase in the LF_n.u._ component of sympathetic modulation of the sino-atrial node and a mild increase in the LF/HF ratio were observed. The gain of the sympathetic baroreflex modulation sBRS was significantly depressed after HDBR (sBRS: −2.9 ± 1.5 burst%/mmHg) compared to before (sBRS: −6.0 ± 1.1 burst%/mmHg). An adequate MSNA signal to noise ratio for burst activity automatic analysis was obtained in 8 of 10 individuals. MSNA was greater after HDBR. The spectral marker of sympathetic vasomotor control, LF_SAP_, was unchanged (Table 2).

During TILT, RR interval, RR variance, and LF_RR_ values were lower after HDBR than before, as well as was the cardiac baroreceptor indices *α*. The cBRS_seq_ index was slightly, but not significantly, lower after HDBR (Table 2).

The gain of sBRS, as indicated by the slope (a) (Figure 1) of the regression line between MSNA-burst % and DAP values, was significantly more depressed after HDBR (sBRS: −2.3 ± 0.7 burst%/mmHg) than before HDBR (sBRS: −4.4 ± 0.4 burst%/mmHg) indicating a less efficient sympathetic baroreflex control after HDBR (Table 2). This pattern was associated with only a slight, although non-significant, increase in MSNA values and a mild decrease in LF_SAP_ during HDBR compared to before HDBR (Table 2). Finally, after HDBR, the effect of orthostatic stimulus on MSNA discharge seems to be blunted, although not significantly, compared to before.

## Discussion

The important finding of our study is that a 3-week lasting HDBR significantly reduced orthostatic tolerance in healthy young men. The response was associated with impaired baroreceptor control of vascular sympathetic drive, both, while supine and during orthostatic testing.

To quantify the potential changes in the orthostatic tolerance induced by HDBR, every volunteer underwent a 80° head-up tilt followed by LBNP (el-Bedawi and Hainsworth, 1994; Protheroe et al., 2013). The approach enabled us to quantify orthostatic tolerance in each individual, although it made MSNA recording procedure more complex. Following HDBR, time to pre-syncope decreased substantially as much less orthostatic stress was tolerated. This observation further indicates that HDBR remarkably impairs orthostatic tolerance.

Chronic bed-confinement is still a common condition in several clinical settings, particularly in patients hospitalized after major trauma and surgery as well as in the elderly (Feldstein and Weder, 2012; Guerin et al., 2016; Tzur et al., 2018). In this context, reduced gravity tolerance, induced by the gravitational and physical deconditioning associated with bed rest, was found to promote an increased risk of loss of consciousness and falls (Shibao et al., 2007; Juraschek et al., 2017).

Several studies addressed the pathophysiological mechanisms potentially underlying the impaired orthostatic tolerance induced by bed rest or after weightlessness (Blomqvist et al., 1980; Pawelczyk et al., 2001; Waters et al., 2005), a condition that is known to mimic the hemodynamic and autonomic effects of the prolonged lying down position. Pathophysiological mechanisms of orthostatic intolerance include hypovolemia induced by central plasma volume redistribution leading to secondary diuresis increase (Iwasaki et al., 2004; Waters et al., 2005), endothelial dysfunction (Coupe et al., 2009), and vascular sympathetic withdrawal (Kamiya et al., 2003). In addition, a proper baroreflex function controlling the cardiovascular system has been highlighted as mandatory for adequate orthostatic tolerance (Robertson et al., 1993; Mosqueda-Garcia et al., 1997; Furlan et al., 2000; Kamiya et al., 2000a,b, 2003; Iwasaki et al., 2004; Heusser et al., 2005; Tank et al., 2012; Marchi et al., 2015). In addition, this study stresses further the relevance to separately assess both cardiac and sympathetic branches of the baroreflex regulatory activity in humans as already reported by previous studies while supine (Dutoit et al., 2010; Taylor et al., 2015) and during incremental head-up tilt (Marchi et al., 2016).

In our study, sympathetic baroreflex control of MSNA in the supine position was substantially attenuated following HDBR. The response was associated with increased MSNA, a finding already described after bed rest (Tanaka et al., 2013) and during (Ertl et al., 2002) and after weightlessness (Levine et al., 2002). Conversely, other authors found a reduced MSNA burst frequency after 14-day HDBR (Shoemaker et al., 1998). Remarkably, SAP and LF_SAP_ were unaffected by HDBR. This observation suggests that an increase in sympathetic activity after HDBR was not sufficient to produce tonic and phasic vasomotor responses.

With regard to the baroreflex control of heart rate, no changes were induced by HDBR when the subjects were supine, in keeping with a previous bed rest study performed under controlled plasma volume conditions (Iwasaki et al., 2004). Conversely, Kamiya and colleagues reported that after 60 and 120 days of HDBR, the gain of the baroreflex control of heart rate was flatter than at baseline (Kamiya et al., 2000a,b). The longer duration of HDBR stimulus compared to the present study may account for the differences on cardiac baroreflex control results. Not surprisingly, in the present study, HR and the spectral indices of RR variability were unmodified after HDBR.

With orthostatic stress, the gain of sympathetic baroreceptor modulation was remarkably depressed, in the presence of only a slight increase in MSNA. This unexpected finding might be accounted for by few possibilities. The statistical power of our study may have been too low to detect MSNA changes. Alternatively, HDBR may have primarily acted on the pre- and/or post-ganglionic sympathetic neurons by blunting their spontaneous discharge activity. If so, reduced baroreceptor inhibition observed after HDBR may not increase post-ganglionic sympathetic firing. Accordingly, SAP and LF_SAP_ were unmodified after HDBR. As expected, the cardiac baroreceptor indexes were lower after HDBR in the presence of a proper HR increase during the gravitational stimulus.

Of interest, Kamiya and colleagues (Kamiya et al., 2000a,b) found that sBRS slopes increased while supine and during 60°HUT, differently from what we observed in the present study. We do believe that a longer duration of HDBR stimulus (60 and 120 compared to our 21 days of HDBR) may account for the differences on both cardiac and sympathetic baroreflex control between our and Kamiya’s results. Indeed, baroreceptor changes following HDBR are likely to be characterized by a time course that could partially explain the observed discrepancies.

In addition, in the present paper, the sBRS was obtained as the relationship between the percentage of times that a MSNA burst was detected as associated with the considered diastolic arterial pressure bin to quantify the sympathetic baroreflex control of the vessels. Such an approach proved to be effective in assessing sympathetic baroreceptor modulation in supine position during a modified Oxford trial (Hart et al., 2010) and during the orthostatic challenge (Barbic et al., 2015; Marchi et al., 2015). Of importance, this index is based on a probabilistic approach that is independent of a normalization procedure. By contrast, other methods enabling the assessment of baroreceptor modulation of sympathetic activity are based on the evaluation of burst amplitude (Kamiya et al., 2000a,b) or area (Doherty et al., 2018) and on their relationship with diastolic arterial pressure changes. Notably, all these approaches require a normalization procedure that in most of the cases is represented by the highest burst observed during baseline condition or during controlled respiration (Diedrich et al., 2009). These normalization procedures are highly dependent on the specific situations occurring during the experimental condition utilized as a reference period and on mathematical procedure utilized to normalize the actual values and specific experimental conditions (Salmanpour and Shoemaker, 2012). As a consequence, any normalization procedure features some degree of arbitrariness.

Our data confirm the usefulness to extend the separate assessment of both cardiac and sympathetic branches of the reflex also when exploring the effects of prolonged physical deconditioning such as after HDBR.

Taken together, our findings suggest that HDBR exerted different changes on cardiac and sympathetic baroreceptor modulation both while supine and during the orthostatic stimulus. In the supine position, cardiac baroreflex gain and HR did not change, whereas a remarkable decrease in sympathetic baroreceptor control with a proper increase of MSNA discharge was evident. During the tilt maneuver, while a proper cardiac baroreceptor gain decrease and tachycardia were found, a concomitant reduction of baroreceptor modulation of sympathetic discharge to the vessels was observed in the absence of appropriate MSNA increase. Therefore, HDBR seems to act mostly on the sympathetic baroreceptor control of vasomotion. Accordingly, our healthy volunteers were unable to tolerate the orthostatic position as much as observed before HDBR.

## Conclusions

Our study confirms the need to separately assess cardiac and sympathetic branches of baroreceptor cardiovascular control. This methodological approach seems to be useful also when exploring the effects of physical deconditioning as mimicked by the medium-term bed rest. Our data bear important implication in clinical setting characterized by a prolonged period of bed confinement and inactivity such as after fractures and major surgery in hospitalized patients and in the elderly. Finally, we hypothesize that baroreceptor assessment might be helpful to identify patients at increased risk of orthostatic intolerance that is more likely to suffer from syncope, falls, and consequent global disability syndrome.

## Ethics Statement

This study was carried out in accordance with the recommendations of the Aerztekammer Nordrhein (Dusseldorf, Germany) with written informed consent from all subjects. All subjects gave written informed consent in accordance with the Declaration of Helsinki. The protocol was approved by the ethic commission of the Aerztekammer Nordrhein (Dusseldorf, Germany).

## Author Contributions

FB and RF were responsible for the conception and design of the study. FB, KH, RF, and PG were responsible for the acquisition of data. FB, BC, MM, DS, and RZ were involved in data analysis. FB, KH, RF, AP, AD, JJ, and JT were involved in data interpretation. FB, RF, KH, JJ, JT, AD, and AP contributed to the drafting of the article. All authors critically revised the draft and approved the final version of the manuscript.

### Conflict of Interest Statement

The authors declare that the research was conducted in the absence of any commercial or financial relationships that could be construed as a potential conflict of interest.
